# MECO: Mixture-of-Expert Codebooks for Multiple Dense Prediction Tasks

**DOI:** 10.3390/s25175387

**Published:** 2025-09-01

**Authors:** Gyutae Hwang, Sang Jun Lee

**Affiliations:** Division of Electronics and Information Engineering, Jeonbuk National University, Jeonju 54896, Republic of Korea; gyutae741@jbnu.ac.kr

**Keywords:** deep learning, computer vision, multi-task learning, vector quantization

## Abstract

Autonomous systems operating in embedded environments require robust scene understanding under computational constraints. Multi-task learning offers a compact alternative to deploying multiple task-specific models by jointly solving dense prediction tasks. However, recent MTL models often suffer from entangled shared feature representations and significant computational overhead. To address these limitations, we propose Mixture-of-Expert Codebooks (MECO), a novel multi-task learning framework that leverages vector quantization to design Mixture-of-Experts with lightweight codebooks. MECO disentangles task-generic and task-specific representations and enables efficient learning across multiple dense prediction tasks such as semantic segmentation and monocular depth estimation. The proposed multi-task learning model is trained end-to-end using a composite loss that combines task-specific objectives and vector quantization losses. We evaluate MECO on a real-world driving dataset collected in challenging embedded scenarios. MECO achieves a +0.4% mIoU improvement in semantic segmentation and maintains comparable depth estimation accuracy to the baseline, while reducing model parameters and FLOPs by 18.33% and 28.83%, respectively. These results demonstrate the potential of vector quantization-based Mixture-of-Experts modeling for efficient and scalable multi-task learning in embedded environments.

## 1. Introduction

Modern autonomous systems such as unmanned vehicles and mobile robots require comprehensive scene understanding in dynamic environments with limited computational resources [1,2]. To achieve robust understanding, recent autonomous systems adopt vision-based deep learning models for dense prediction tasks, including semantic segmentation [3] and monocular depth estimation [4]. The development of large-scale foundation models [5,6] has significantly improved the performance of vision-based models, while also increasing computational requirements. However, most mobility platforms are equipped with limited computing resources due to power and manufacturing cost constraints, making it challenging to deploy large-scale models directly. Specifically, embedded environments such as rural or mountainous regions cannot rely on real-time cloud services due to unstable communication infrastructure [7]. This requirement is in contrast to the need for multiple task-specific models and large-scale architectures to enable comprehensive scene understanding [8].

Multi-task learning (MTL) aims to jointly learn multiple tasks from a single input image by leveraging diverse supervision to enhance scene understanding [9]. Pixel-level prediction, often referred to as dense prediction, includes downstream tasks such as segmentation, depth estimation, optical flow estimation, and surface normal prediction. These tasks typically adopt encoder–decoder architectures to match the spatial resolution between the input image and the visual output. To reduce computational redundancy, hard parameter sharing-based MTL approaches employ a unified architecture that shares a single encoder across tasks while using task-specific decoders [10]. However, the shared encoder often produces entangled feature representations—also referred to as negative transfer—where task-specific and task-agnostic information are mixed, hindering task-wise optimization [11]. Existing MTL architectures can be broadly categorized into encoder-focused [12,13] and decoder-focused [14,15] designs, both of which struggle to fully disentangle shared representations [16]. In contrast, MoE-based architectures [17,18] enhance latent disentanglement by explicitly partitioning representations into task- or domain-specific subspaces, enabling each expert to specialize and improving generalization to dense prediction tasks [19].

Among various approaches in MTL, Mixture-of-Experts-based methods offer a promising solution for improving computational efficiency and disentangling feature representations by enhancing model sparsity. Mixture-of-Experts (MoE) [20] arranges multiple expert networks in parallel and selectively activates the most relevant expert routes for a given input feature map. Recent MTL models [18,21] have leveraged task-specific routers and multiple shared expert networks to efficiently disentangle task features from encoder representations. While these methods have advanced feature disentanglement and task-specific routing, they still rely on computationally heavy expert networks that limit their applicability in resource-constrained environments. These limitations highlight the need for a lightweight alternative, such as vector quantization-based experts. Moreover, deploying a large-scale MTL model on low-power embedded devices requires multiple rounds of compression techniques such as knowledge distillation [22], pruning [23], and quantization [24]. To perform progressive compression while retaining the prior knowledge of the initial large-scale model, fine-tuning at each stage is essential. Multiple fine-tuning steps incur substantial time and computational overhead, making it crucial to propagate both task-generic and task-specific representations of the initial model.

In this paper, we propose Mixture-of-Expert Codebooks (MECO), a multi-task learning framework that leverages quantized codebooks as a Mixture-of-Experts for multiple dense prediction tasks. Vector quantization (VQ) [25] transforms the continuous latent space of a model into a finite set of discrete vectors (codebook), enabling compact representations and efficient memory access. In MECO, each expert codebook operates as a subset of the latent space, disentangling and routing the encoder representation into task-specific features. Since the transformation of task-specific features is performed via Euclidean distance-based codebook lookup, it significantly reduces memory overhead. However, as Euclidean distance in high-dimensional spaces may suffer from the curse of dimensionality, we jointly learn the codebook and latent features to preserve its discriminative power in the embedding space. In the context of model compression, fixed expert codebooks learned from a large-scale encoder can be transferred to fine-tuning steps, facilitating rapid convergence in compressed models.

We introduce a method for training expert codebooks from a large-scale MTL model and evaluate its effectiveness on real-world driving datasets collected in embedded environments. Our main contributions are summarized as follows.

- We propose an end-to-end multi-task learning framework that learns task-generic and task-specific codebooks via vector quantization, disentangling shared representations into a discrete latent space for task-specific features
- We introduce a Mixture-of-Expert Codebooks design that substantially improves computational efficiency by replacing multiple expert modules in MoE with lightweight codebooks
- We present a real-world driving dataset for embedded environments, enabling evaluation of the proposed method’s efficiency gains and maintained task performance over prior approaches

The rest of this paper is organized as follows. Section 2 presents related work. Section 3 explains the proposed MTL model architecture and loss functions for multiple dense prediction tasks. Section 4 and Section 5 present experimental results and conclusions.

## 2. Related Work

### 2.1. Multi-Task Learning

Multi-task learning is a unified model to learn multiple related tasks simultaneously by sharing intermediate representations, improving generalization and reducing redundancy. Recent studies have explored the use of intermediate modules between the encoder and task decoders to better disentangle shared representations into task-specific components. Inverted Pyramid multi-task Transformer (InvPT) [26] uses parallel preliminary task decoders to generate task-specific features, followed by a multi-task UP-Transformer block that fuses them in a coarse-to-fine manner, enhancing cross-task interaction but increasing computational and memory overhead. TaskExpert [21] utilizes MoE to decompose backbone representation and incorporates a multi-task feature memory as an additional expert to extract long-range task features from hierarchical encoder layers. However, as the number of experts increases, computational overhead becomes significant. To address this limitation, Mixture of Low-rank Experts (MLoRE) [18] replaces full experts with low-rank adaptation (LoRA) modules, preserving much of MoE’s disentanglement ability while improving efficiency. However, applying low-rank experts across multiple backbone layers still incurs non-trivial computational cost in resource-constrained settings. While MoE provides strong disentanglement of shared representations, applying it across multiple backbone layers still results in considerable computational cost.

### 2.2. Mixture-of-Experts

Mixture-of-Experts models ensemble the outputs of multiple specialized subnetworks via a routing mechanism, enabling sparse and adaptive computation. A notable study [20] proposed a top-*k* based MoE approach that significantly increases model capacity relative to computational cost, contributing to advancements in natural language processing. In detail, the router network transforms the semantic information of the input features into a weight vector and executes computations only on the top-*k* weighted experts. In the field of MTL, Ma et al. proposed Multi-gate Mixture-of-Experts (MMoE) [17] to reduce task interference and aggregate task-specific features through task-wise router networks. Recently, WEMoE [27] has been proposed as a layer-wise MoE framework that transforms modules from single-task models into experts and uses a learned router for input-dependent weight aggregation. However, it is limited by the requirement for trained single-task models, which restricts its applicability in scenarios where such models are unavailable or costly to obtain. Inspired by prior MoE approaches, the proposed MECO module employs a top-*k* and task-wise routing strategy from a shared encoder representation to handle multiple dense prediction tasks. To significantly reduce computational cost and compress disentangled representations from experts, we integrate VQ into the MoE framework.

### 2.3. Vector Quantization

Vector quantization [28] is a technique that reconstructs continuous high-dimensional vectors into a finite set of vectors, known as a codebook. In deep neural networks, VQ discretizes continuous feature representations by mapping them to the nearest codebook entries to facilitate compact representation learning. Oord et al. proposed the Vector Quantized-Variational Autoencoder (VQ-VAE) [25], which learns discrete latent representations that enable high-quality image reconstruction and efficiently extend to generative modeling. However, VQ can suffer from quantization error that reduces representation quality and codebook collapse where only a subset of codewords are utilized. To mitigate these issues, they proposed a loss function based on stop-gradient and the straight-through estimator [29]. It incorporates commitment loss to align codewords with encoder outputs and diversity regularization to encourage uniform codebook usage. Moreover, VQ compresses features into indices, reducing memory usage and enabling fast codebook access during inference. In addition, VQ-Prompt [30], which has achieved high performance in the field of class-incremental continual learning, represents task-specific prompts as discrete codes via vector quantization and optimizes them end-to-end. Inspired by prior work, the proposed MECO leverages multiple discretized codebooks to disentangle representations into highly relevant quantized vectors and trains the entire framework in an end-to-end manner.

## 3. Method

In this section, we present the architecture of the proposed MTL model and MECO module, and it is organized as follows. Section 3.1 summarizes the baseline MoE frameworks for MTL. Section 3.2 illustrates the overall architecture of the proposed MTL model. Section 3.3 and Section 3.4 elaborates on the MECO module and task-specific vector quantization, and Section 3.5 defines the loss functions to jointly optimize the proposed model and its codebooks. We have summarized the notations and subscripts used in this section in Table 1.

### 3.1. Preliminaries: Mixture-of-Experts

Before introducing our proposed MECO-based MTL model, we first review the standard MoE architecture [20] and its variants used in MTL. Let $x\in R^{M\times D}$ be the input feature representation, where *M* and *D* denote the number of tokens and the latent dimension, respectively. An MoE model consists of a router network $f_{r}\left(\cdot \right)$ and a set of *N* expert networks, denoted as $E=\{E_{1},\ldots ,E_{N}\}$. The router network computes a weight vector $w\in R^{N}$, assigning higher values to the most relevant experts based on the semantic features of *x*. Each expert is implemented as a fully connected layer that independently extracts features from the input *x*. The outputs of the experts are aggregated through a weighted summation as a linear combination with the router weights *w* and can be formulated as follows:

(1) $$U=f_{\mathrm{moe}}\left(x\right)=\sum_{n=1}^{N}w_{n}\cdot E_{n}\left(x\right),\;f_{r}\left(x\right)=w.$$

where *U* denotes the final output of the MoE $f_{\mathrm{moe}}\left(\cdot \right)$, with $w_{n}$ representing the scalar weight for expert $E_{n}$.

To extend MoE to MTL, the Multi-gate Mixture-of-Experts framework [17] was proposed to extract task-specific features from shared experts. MMoE modifies the standard MoE by introducing task-specific routers $f_{r,t}\left(\cdot \right)$ for each task t={1,…,T}, allowing the model to learn distinct routing strategies for different tasks. Subsequently, the Mixture of Low-rank Experts [18] was proposed, which applies LoRA to experts in order to reduce their memory overhead. In MLoRE, the input embeddings are first permuted into spatial representations $x\in R^{D\times H^{\prime }\times W^{\prime }}$ and then passed through task-specific feature extractors $f_{e,t}\left(\cdot \right)$ implemented as a 1×1 convolution. Each expert is composed of a low-rank bottleneck structure with rank *r* to reduce the number of trainable parameters and computational cost and is expressed as follows:

(2) $$E_{n}\left(x\right)=B_{n}\left(A_{n}\left(x\right)\right),$$

where $A_{n}\in R^{3\times 3\times D\times r}$ and $B_{n}\in R^{1\times 1\times r\times D}$ denote 3×3 and 1×1 convolutions, respectively, and r≪D. In this case, the multiply–accumulate operations (MACs) for each expert are computed as $H^{\prime }\times W^{\prime }\times r\times D\times \left(3^{2}+1\right)$. The MLoRE module $f_{\mathrm{mlore}}\left(\cdot \right)$ produces the final output for task *t*, defined as follows:

(3) $$U_{t}=f_{\mathrm{mlore}}\left(x\right)=\sum_{n=1}^{N}w_{t,n}\cdot E_{n}\left(f_{e,t}\left(x\right)\right),\;f_{r,t}\left(x\right)=w_{t}.$$

### 3.2. The MTL Model Architecture

**Figure 1:**
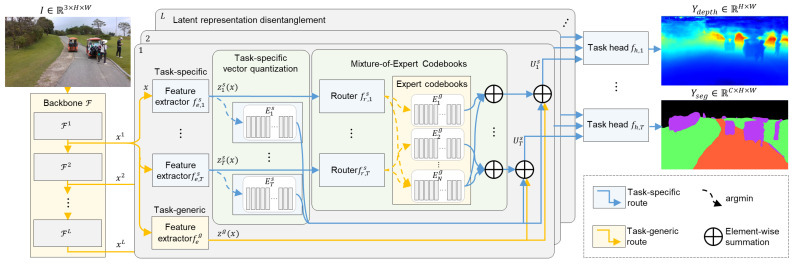
Architecture of the proposed MTL model.

The proposed MTL model is based on the MMoE framework and incorporates the MECO to disentangle latent representations, as illustrated in Figure 1. The overall architecture consists of three major components: a backbone network, latent representation disentanglement, and task-specific heads. First, we adopt a Vision Transformer (ViT) [31] backbone since its ability to model long-range dependencies facilitates learning shared representations across tasks [32,33]. The ViT backbone with *L* layers takes an RGB image $I\in R^{3\times H\times W}$ as input, where *H* and *W* denote the height and width, respectively. Second, the latent representation disentanglement stage processes multi-scale features extracted from each ViT layer l∈{1,…,L} and consists of the MECO module $f_{\mathrm{meco}}\left(\cdot \right)$, task-specific vector quantization $f_{\mathrm{specific}}\left(\cdot \right)$, and a task-generic route $f_{\mathrm{generic}}\left(\cdot \right)$. Finally, each task-specific head $f_{h,t}\left(\cdot \right)$ generates a dense prediction $Y_{\mathrm{task}}$, which can be formulated as follows:

(4) $$Y_{\mathrm{task}}=f_{h,t}\left(\sum_{l=1}^{L}\left(f_{\mathrm{meco}}\left(F^{l}\left(I\right)\right)+f_{\mathrm{specific}}\left(F^{l}\left(I\right)\right)+f_{\mathrm{generic}}\left(F^{l}\left(I\right)\right)\right)\right),$$

where $F^{l}\left(I\right)$ denotes the intermediate representation of the *l*-th backbone layer.

Latent representation disentanglement aims to decompose the intermediate representation $F^{l}\left(I\right)=x^{l}\in R^{D\times H^{\prime }\times W^{\prime }}$ through three distinct routes. Note that t∈{1,…,T} and n∈{1,…,N} denote the task and expert indices, and *s* and *g* indicate task-specific and task-generic components, respectively. For clarity, we omit the backbone layer index *l* in what follows. The feature extractors ${\left\{f_{e,t}^{s}\left(\cdot \right)\right\}}_{t=1}^{T}$ and $f_{e}^{g}\left(\cdot \right)$, implemented as 1×1 convolutions, compute the *T* task-specific representations $z_{t}^{s}\left(x\right)$ and a task-generic representation $z^{g}\left(x\right)$, respectively. Generally, the representation z(x) is defined as

(5) $$z\left(x\right)=f_{e}\left(x\right).$$

The first and second routes, $f_{\mathrm{meco}}\left(\cdot \right)$ and $f_{\mathrm{specific}}\left(\cdot \right)$, take $z_{t}^{s}\left(x\right)$ as input and map into quantized latent spaces using codebooks that represent task-generic and task-specific semantics. The third route, $f_{\mathrm{generic}}$, directly passes $z^{g}\left(x\right)$ as a residual connection, preserving generic semantic information. This residual path also mitigates potential information loss from VQ by providing an uncompressed representation to the task-specific head. The disentangled representations from the three routes are fused into a task-specific feature $U_{t}^{s}$, computed as follows:

(6) $$U_{t}^{s}=\sum_{n=1}^{N}w_{t,n}\cdot {\mathrm{Quantize}}_{n}\left(z_{t}^{s}\left(x\right)\right)+{\mathrm{Quantize}}_{t}\left(z_{t}^{s}\left(x\right)\right)+z^{g}\left(x\right),\;f_{r,t}^{s}\left(z_{t}^{s}\left(x\right)\right)=w_{t},$$

where ${\mathrm{Quantize}}_{n}\left(\cdot \right)$ and ${\mathrm{Quantize}}_{t}\left(\cdot \right)$ represent VQ function using the *n*th expert codebook and the task-specific codebook, respectively. We applied batch normalization after the MECO term and the summation to ensure training stability and scale consistency.

### 3.3. Mixture-of-Expert Codebooks

**Figure 2:**
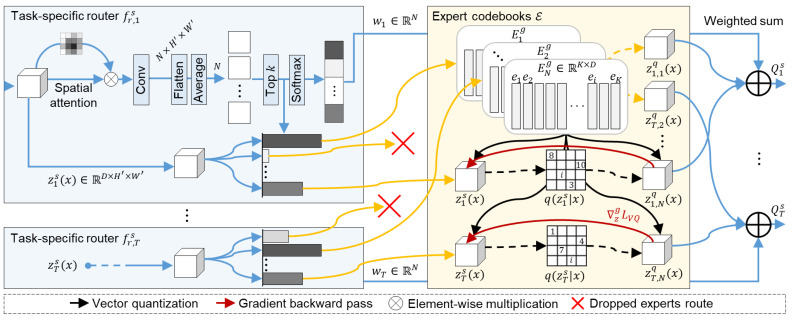
Architecture of the proposed MECO module.

The MECO module is designed to extract task-specific features from *N* task-generic expert codebooks. The detailed architecture is illustrated in Figure 2. MECO follows a top-*k* MoE structure, consisting of task-specific routers ${\left\{f_{r,t}^{s}\left(\cdot \right)\right\}}_{t=1}^{T}$ and a set of expert codebooks $E={\left\{E_{n}^{g}\right\}}_{n=1}^{N}$. The task-specific router takes the task-specific representation $z_{t}^{s}\left(x\right)$ as input and produces a weight vector $w_{t}\in R^{N}$. For each selected expert index *n*, VQ is performed on $z_{t}^{s}\left(x\right)$ using the corresponding expert codebook $E_{n}^{g}$. Each expert codebook defines an independently distributed discrete latent space that is shared across tasks, promoting the disentanglement of latent representations. Finally, the task-specific feature $Q_{t}^{s}$ is computed as a weighted summation of the quantized outputs from the *k* selected expert codebooks, using the weights $w_{t}$:

(7) $$Q_{t}^{s}=\sum_{n\in K}w_{t,n}\cdot {\mathrm{Quantize}}_{n}\left(z_{t}^{s}\left(x\right)\right),$$

where K denotes the set of top-*k* selected indices. Note that this is expressed differently from the first term in Equation (6), as the dropped experts are masked out from the *N* experts.

A task-specific router processes the task-specific representation $z_{t}^{s}\left(x\right)\in R^{D\times H^{\prime }\times W^{\prime }}$ to compute the top-*k* expert selections along with their corresponding router weights $w_{t}$. Spatial attention highlights important regions in the representation, which is then restructured into a condensed form for expert selection. This is followed by a 1×1 convolution, flattening, and spatial average pooling to produce task logits $\omega_{t}\in R^{N}$ for each expert codebook. The top-*k* function retains the *k* largest logits and masks the dropped logits with −∞, which can be written as follows:

(8) $${\tilde{\omega }}_{t,n}=\left\{\begin{array}{cc} \omega_{t,n}, & \mathrm{if}\;n\in K,\\ -\infty , & \mathrm{otherwise} \end{array}\right..$$

where ${\tilde{\omega }}_{t}$ denotes the masked logit vector for task *t*, which is subsequently normalized by softmax to produce a probability distribution:

(9) $$w_{t,n}=\frac{\exp({\tilde{\omega }}_{t,n})}{\sum_{m\in K}\exp\left({\tilde{\omega }}_{t,m}\right)}.$$

The router activates only the selected top-*k* experts during the forward pass, thereby reducing the computation of the dropped routes and promoting sparsity. This conditional execution mechanism ensures efficiency by selectively activating task-relevant experts, leading to reduced resource consumption in both training and inference.

Expert codebooks quantize the representations by mapping them to the closest entries in the *k* codebooks selected by the task-specific router. Given the task-specific representation $z_{t}^{s}\left(x\right)\in R^{D\times H^{\prime }\times W^{\prime }}$, we express it as a set of latent vectors ${\left\{z_{m}\right\}}_{m=1}^{M}$ for simplicity, where $z_{m}\in R^{D}$ and $M=H^{\prime }\times W^{\prime }$. Each expert codebook $E_{n}^{g}\in R^{K\times D}$ contains *K* codewords ${\left\{e_{i}\right\}}_{i=1}^{K}$, with $e_{i}\in R^{D}$ representing discretized latent vectors. For the selected expert codebook, VQ is performed by a nearest neighbor lookup between $z_{m}$ and ${\left\{e_{i}\right\}}_{i=1}^{K}$, which can be formulated as follows:

(10) $$z_{m}^{q}=e_{i},\;\mathrm{where}\;i=\arg\min_{j}{∥z_{m}-e_{j}∥}_{2},$$

where the superscript *q* in $z_{m}^{q}$ denotes the quantized version of the latent vector $z_{m}$, and the index *j* refers to codewords used for nearest neighbor search. This hard assignment can be represented as the following one-hot posterior categorical distribution:

(11) $$q\left(z_{m}=e_{i}|x\right)=\left\{\begin{array}{cc} 1 & \mathrm{if}\;i=\arg\min_{j}{∥z_{m}-e_{j}∥}_{2},\\ 0 & \mathrm{otherwise} \end{array}\right.,$$

where $q(z_{m}=e_{i}|x)$ denotes the deterministic probability that the latent vector $z_{m}$, extracted from input *x*, is hard-assigned to the codeword $e_{i}$. Using the quantized results $z_{m}^{q}$ from all *M* positions, we define the VQ function for *n*th expert codebook ${\mathrm{Quantize}}_{n}\left(\cdot \right)$ as follows:

(12) $$z_{t,n}^{q}\left(x\right)={\mathrm{Quantize}}_{n}\left(z_{t}^{s}\left(x\right)\right)=\mathrm{Concat}{\left(z_{t,n,m}^{q}\right)}_{m=1}^{M},$$

where $z_{t,n}^{q}\left(x\right)\in R^{D\times H^{\prime }\times W^{\prime }}$ denotes the quantized representation for task *t* and expert *n*, and Concat(·) refers to the operation that aggregates the quantized latent vectors according to their spatial positions. The MACs for codebook lookup are computed as $H^{\prime }\times W^{\prime }\times K\times D$ based on the dot product between *z* and *e*. Accordingly, VQ has a lower computational cost than LoRA when K<10r. MLoRE employs a range of expert ranks from 16 to 240 with the step of 16. In our experiments with K=256, VQ reduces per-expert computation by 20% to 89% in all cases except when r=16.

### 3.4. Task-Specific Vector Quantization

Task-specific vector quantization is designed to learn a task-specialized discrete latent space represented by $E_{t}^{s}\in R^{K\times D}$. Following the VQ mechanism of the expert codebook, each task codebook quantizes the task-specific representation $z_{t}^{s}\left(x\right)$ and follows the same formulation as in Equations (10) and (11). The VQ function for the *t*th task codebook, ${\mathrm{Quantize}}_{t}\left(\cdot \right)$, is formulated as follows:

(13) $${\mathrm{Quantize}}_{t}\left(z_{t}^{s}\left(x\right)\right)=\mathrm{Concat}{\left(z_{t,m}^{q}\right)}_{m=1}^{M}.$$

To complement the features necessary for solving each task, task codebooks construct a distinct quantized latent space that is separated from the task-generic MECO to facilitate the disentanglement of task-specific representations.

### 3.5. Loss Function

To jointly optimize task performance and discrete representation learning in an end-to-end manner, we define a composite loss function consisting of VQ losses and dense prediction task-specific losses. Since the argmin operation used in VQ is non-differentiable, the loss $L_{\mathrm{VQ}}$ is decomposed into two terms: a dictionary loss and a commitment loss. Both losses are defined based on the L2 norm between the task-specific representation $z_{m}$ and the quantized representation $e_{i}$. The dictionary loss updates the codebook by stopping gradients from $z_{m}$, whereas the commitment loss updates the feature extractor $f_{e}\left(\cdot \right)$ by stopping gradients from $e_{i}$, as follows:

(14) $$L_{\mathrm{VQ}}=\underset{\;\mathrm{Dictionary}\;\mathrm{loss}}{\underset{︸}{∥\mathrm{sg}\left[z_{m}\right]-e_{i}{∥}_{2}^{2}}}+\beta \underset{\mathrm{Commitment}\;\mathrm{loss}}{\underset{︸}{∥z_{m}-\mathrm{sg}\left[e_{i}\right]{∥}_{2}^{2}}},$$

where sg[·] denotes the stop-gradient operator, and β is a weighting factor that balances the commitment loss. The dictionary loss encourages each expert and task codebook to learn task-generic and task-specific latent spaces, respectively. The commitment loss encourages ask-specific representation to stay close to their assigned codewords, promoting consistent codeword usage and preventing under-utilization that can lead to codebook collapse. During backpropagation, the straight-through estimator is adopted to address the non-differentiability of VQ. It approximates gradients by directly copying them from the quantized vector $e_{i}$ to the extractor output $z_{m}$. By jointly optimizing these two VQ losses, the codebook and feature extractor align the learned embedding space, thereby mitigate the risk of the curse of dimensionality.

The total loss is composed of the expert codebook VQ loss ($L_{\mathrm{VQ}}^{g}$), the task codebook VQ loss ($L_{\mathrm{VQ}}^{s}$), and the dense prediction task losses. It is defined as follows:

(15) $$L_{\mathrm{total}}=\sum_{t=task}\left(\sum_{n\in K}L_{\mathrm{VQ}}^{g}+L_{\mathrm{VQ}}^{s}+\lambda_{t}L_{t}\right),$$

where both $L_{\mathrm{VQ}}^{g}$ and $L_{\mathrm{VQ}}^{s}$ are the sum of $L_{VQ}$ over $M=H^{\prime }\times W^{\prime }$ spatial locations. $L_{\mathrm{VQ}}^{g}$ is computed over the set of expert codebooks K selected for task *t*, and $\lambda_{t}$ denotes the weighting factor for the corresponding task loss.

## 4. Results

### 4.1. Real-World Driving Dataset

**Figure 3:**
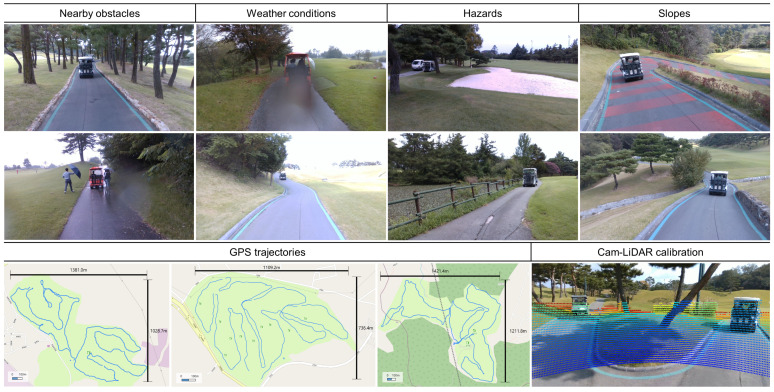
Overview of a real-world driving dataset collected in mountainous golf courses, illustrating diverse conditions (**top**), GPS trajectories (**bottom left**), and camera–LiDAR calibration (**bottom right**).

We collected a real-world driving dataset for the development of visual perception and collision avoidance systems in embedded environments, as illustrated in Figure 3. The data was acquired from driving scenarios within three different mountainous golf courses. The golf course poses unique challenges with narrow roads and nearby obstacles such as trees and fences. This driving scene also features slopes, uneven surfaces, and sand or water hazards uncommon in typical road scenes. These hazard regions are challenging due to their irregular appearance and semantic ambiguity. Furthermore, the vehicle is equipped with a compact embedded system, necessitating lightweight models capable of real-time image processing. As a result, there is a demand for efficient deep learning models that can perform dense prediction tasks, such as monocular depth estimation and semantic segmentation, under real-world constraints. The objective of this research is to develop a large-scale teacher model as an intermediate step toward a lightweight MTL framework optimized for deployment on embedded platforms.

The data collection platform consists of a multi-sensor system including a Intel RealSense D435i RGB-D (Intel, Santa Clara, USA) camera, an Ouster OS1 (Ouster, San Francisco, USA) light detection and ranging (LiDAR), GPS, and an NVIDIA Jetson AGX Orin (NVIDIA, Santa Clara, USA). All sensors are synchronized via the robot operating system (ROS), and front-view driving data is recorded at 10 Hz. To ensure environmental diversity, the data was collected across various seasons and weather conditions, including scenarios with preceding vehicles and multiple pedestrians. The camera and LiDAR were installed to capture RGB images at a resolution of 1280 × 720 and corresponding 3D point clouds. Sparse ground-truth depth maps were obtained using a camera–LiDAR calibration [34]. Semantic segmentation includes five classes: background, fairway, road, hazard, and obstacle. Hazards refer to regions such as lakes and sand traps, while obstacles include humans, vehicles, trees, and signposts.

Annotation of semantic segmentation task involves two main challenges. First, there is the rarity of critical hazard objects; classes such as person and hazard appear infrequently in driving scenes. Second, the pixel-level annotation cost is substantial, as it requires manual refinement of meaningful regions for each image. To address the limitations of constructing the segmentation dataset, we curated the training and test sets according to the following protocol: (1) Maintained a clear separation of driving sequences between the training and test sets. (2) Included data with varying weather conditions and pedestrian scenarios. (3) Incorporated hazard classes, such as bunkers and lakes, which are rare in the driving scene. (4) Selected samples maintaining a uniform distribution of distance to preceding vehicles. Initial semantic labels were generated using the Segment Anything Model (SAM) [5], followed by manual refinement for accuracy. Finally, the images, ground truth depth maps, and semantic labels for training and testing consist of 355 and 96 samples, respectively.

### 4.2. Experiment Environments

Experiments were conducted on a workstation equipped with an Intel Core i9-10940X CPU, 64 GB DDR4 RAM, and dual NVIDIA GeForce RTX 3090 Ti GPUs. The proposed MTL model was implemented using Python 3.7 and PyTorch 1.10. To reduce computational costs and enable further model compression, both input RGB images and ground-truth annotations were resized to a resolution of 640 × 352 during training and evaluation. We used the Adam optimizer with a learning rate of $1\times 10^{-5}$ and a weight decay of $1\times 10^{-6}$. The batch size was set to 2 for all experiments. Due to the small dataset size and batch size, we set the number of training epochs to 500 and report the quantitative results of the model that achieved the best average performance across tasks. The ViT backbone used in the proposed model was initialized with weights pretrained on the ImageNet-1K dataset for 20K training steps. The codebooks were initialized using a uniform random distribution in the range $U(-\sqrt{3},\sqrt{3})$, which ensures each embedding vector starts with equal variance and avoids bias toward any particular direction in the embedding space. As a baseline, we adopted Mixture of Low-rank Experts, which was trained under the same experimental settings.

### 4.3. Hyperparameters and Evaluation Metrics

The proposed model incorporates several hyperparameters related to the MoE framework and VQ. Both the number of experts and the top-*k* selection parameter were set to 15 and 9, respectively, consistent with the MLoRE baseline. These settings are maintained in our main experiments, and their detailed effects are discussed in the ablation study (Section 4.6). The number of latent vectors in each expert codebook was set to K=256. The commitment loss weighting factor β=0.25 was adopted following common VQ-VAE practice, balancing the commitment and dictionary losses while ensuring stable codebook updates. For semantic segmentation, we report the mean intersection over union (mIoU) as the primary evaluation metric, along with class-wise IoU. For monocular depth estimation, we adopt four commonly used error metrics: root mean squared error (RMSE), RMSE *log*, absolute relative error (Abs Rel), and squared relative error (Sq Rel). To assess model efficiency, we report the number of parameters (#Params), floating point operations (FLOPs), and frames per second (FPS) as computational cost indicators.

### 4.4. Experimental Results on Real-World Dataset

To evaluate the effectiveness of the proposed MTL model on a real-world driving dataset, we conducted a quantitative analysis. Table 2 presents the test results of both single-task and the MTL models on the dense prediction tasks of depth estimation and semantic segmentation. In general, MoE-based MTL models outperform their single-task counterparts and demonstrate the advantage of producing multiple task outputs with a unified model. The Mamba-based MTL models generally exhibit lower performance, showing inferior results in both depth estimation and semantic segmentation compared to the MoE-based MTL models. This suggests that the MoE-based approach is more robust to negative transfer by deriving task-specific interpretations of the latent representation from experts. Compared to the baseline MLoRE, the proposed MECO achieves a 0.4% higher mIoU in semantic segmentation. Additionally, MECO shows improved performance across all semantic classes except for the background, while maintaining depth estimation performance close to that of the baseline. These results suggest that the codebooks disentangle representations, thereby enhancing classification performance through distinct latent features.

### 4.5. Computational Complexity

Table 3 compares the computational complexity of the baseline model and the proposed MTL model. By replacing expert modules with codebook-based VQ, MECO achieves significant efficiency gains over the LoRA-based baseline. Specifically, MECO reduces the number of parameters and FLOPs by 18.33% and 28.83%, respectively, compared to MLoRE. The large reduction in FLOPs is attributed to the nature of VQ, where the representation is obtained through an argmin lookup operation rather than dense matrix multiplication, as in LoRA. Moreover, real-time inference evaluation under our experimental setup shows a practical improvement of 3.59 FPS. These results demonstrate that VQ enables substantial model compression with minimal compromise in performance.

### 4.6. Ablation Study

We conduct an ablation study to investigate the effectiveness of model architecture and hyperparameters on performance. Table 4 reports the quantitative performance and computational complexity with respect to the number of expert codebooks *N* in MECO. The number of activated experts was set to 60% of *N* using top-*k* routing, following the baseline configuration to ensure a fair comparison. As the number of experts increases, we observe consistent improvements in both segmentation performance and depth estimation accuracy. Notably, the FLOPs remain nearly constant, indicating that VQ does not significantly contribute to the actual computational load during inference. Since the codebook vectors are learnable parameters, the total number of parameters increases proportionally with the number of experts *N*.

Table 5 presents the performance of the proposed model on dense prediction tasks under different routing strategies and codebook update mechanisms. The proposed MECO model aims at lightweight design through vector quantization, and the routing strategy and codebook update method are selected based on empirical experimental results. Soft routing refers to a probabilistic mixture of expert outputs, where all experts are partially activated with weights derived from a softmax distribution. Experimental results show that the performance gap in semantic segmentation between the two routing strategies is merely 0.025%, whereas the difference in depth estimation reaches approximately 8%, demonstrating the superiority of top-*k* routing for this task. In the terms of model efficiency, top-*k* routing is also more appropriate than soft routing, as it activates only a subset of experts rather than all experts. In soft routing all experts receive non-zero weights so even task-irrelevant experts contribute to the output. These low-weight but irrelevant contributions can introduce noise into the aggregated features and lead to degraded performance, especially in tasks such as depth estimation where feature precision is critical.

On the other hand, the EMA-based method updates the codebook vectors using an exponential moving average (EMA) of encoder outputs, rather than optimizing them as learnable parameters. Since EMA does not require backpropagation through the codebook, the total number of parameters in MECO is reduced to 389.406 M. However, EMA does not contribute to improving inference speed, and its performance across all tasks is inferior to that of the dictionary loss-based approach. The performance gap arises from EMA not directly optimizing encoder–codeword alignment, leading to slower adaptation and less task-specific specialization.

### 4.7. Qualitative Results

**Figure 4:**
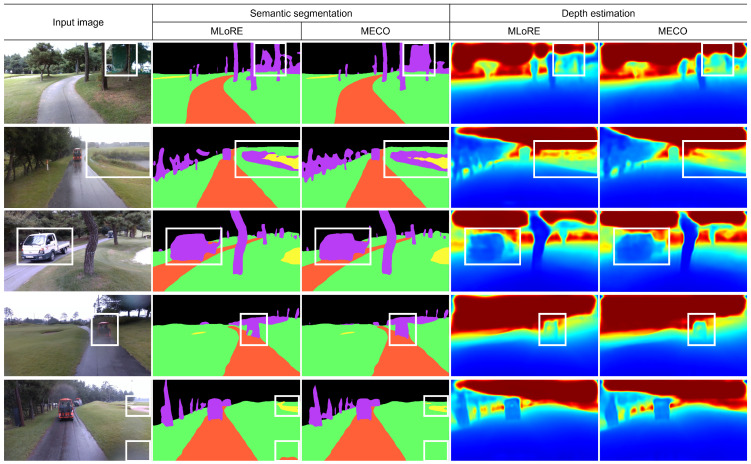
Visualization of the semantic segmentation and depth estimation results.

Figure 4 presents qualitative comparisons between the proposed MTL model and the baseline model across a set of challenging cases. Each column shows the input RGB image, semantic segmentation mask, and predicted depth map, where task predictions are shown for both the baseline and MTL models. We visualized cases involving semi-transparent regions, extremely rare object (truck), distant hazards, and occlusion caused by raindrops. Regions of interest are highlighted using white bounding boxes to emphasize critical areas for comparison. In Case 1, MECO demonstrates superior segmentation of semi-transparent obstacle regions, accurately capturing their boundaries and shapes. In Case 2, MECO produces more consistent and spatially coherent depth estimates in visually ambiguous landscapes. In Case 3, both the segmentation mask and depth map of a truck show greater consistency in MECO’s output compared to the baseline, suggesting better extraction of task-generic representations. Cases 4 and 5 illustrate rainy driving conditions with image blur, where MECO demonstrates robustness by maintaining high prediction quality under low visibility.

## 5. Conclusions

In this paper, we presented Mixture-of-Expert Codebooks, a novel multi-task learning framework that leverages vector quantization for efficient dense prediction in embedded environments. MECO introduces expert codebooks as discrete latent spaces, which effectively disentangle task-specific and task-generic features with minimal computational overhead. By replacing traditional expert networks with quantized codebooks, the proposed model significantly reduces computational costs while maintaining competitive performance across tasks. Our experiments on a real-world driving dataset demonstrate that MECO achieves improved segmentation accuracy and depth estimation performance close to that of the baseline model. Additionally, MECO shows strong robustness under challenging conditions such as low visibility and ambiguous landscapes. However, MECO’s generalizability may be limited by the dataset size, modest performance gains, and lack of external benchmarks. We plan to address these issues through greater dataset diversity, improved robustness, and external evaluations. We also aim to explore a wider range of dense prediction tasks, such as instance segmentation and surface normal estimation. Furthermore, we will investigate deployable model compression techniques—including student model distillation and quantization-aware training—leveraging expert codebooks pretrained on large-scale MTL models.

## Figures and Tables

**Table 1:** List of notations used in this paper.

Notation	Description	Notation	Description	Notation	Description	Notation	Description
*I*	Input RGB image	*t*	Task index	*h*	Task-specific head	*n*	Expert index
*Y*	Dense prediction output	*s*	Task-specific component	*U*	Aggregated features	*K*	Number of codewords
H,W	Height and width of the input image	*g*	Task-generic component	*Q*	Aggregated VQ features	$e_{i}$	*i*-th codeword
$H^{\prime },W^{\prime }$	Height and width of the feature map	*q*	Quantized vector	z(x)	Feature representation	*w*	Router weight vector
*M*	Number of tokens ($M=H^{\prime }\times W^{\prime }$)	*L*	Number of ViT layers	*z*	Latent vector	*k*	Number of top-*k* selection
*m*	Spatial position index (VQ)	*l*	ViT layer index	${\mathrm{Quantize}}_{n}\left(\cdot \right)$	VQ function of expert codebook	K	Set of selected expert indices
*D*	Feature dimension	*f*	Functions of modules	${\mathrm{Quantize}}_{t}\left(\cdot \right)$	VQ function of task codebook		
L	Loss function	*e*	Extractor	*E*	Codebook		
*T*	Number of tasks	*r*	Task-specific router	*N*	Number of experts		

**Table 2:** Depth estimation and semantic segmentation performance on the real-world driving dataset. ↓ and ↑ denote whether a lower or higher value is better for each task, respectively.

Task	Method	Depth Estimation ↓				Semantic Segmentation ↑					
		RMSE	RMSE Log	Abs Rel	Sq Rel	Background	Fairway	Hazard	Road	Obstacle	mIoU (%)
Single	BTS [35]	2.758	0.195	0.129	0.541	-	-	-	-	-	-
	UNetFormer [36]	-	-	-	-	82.37	93.86	65.79	95.83	52.61	78.09
Multiple	MTMamba [37]	2.185	0.181	0.115	0.453	82.99	93.12	50.66	94.87	54.21	75.17
	MTMamba++ [38]	2.850	0.210	0.129	0.579	84.03	93.93	56.31	95.56	55.46	77.06
	MLoRE [18]	2.198	0.157	0.101	0.358	85.65	94.16	61.81	96.50	59.99	79.63
	MECO (ours)	2.224	0.159	0.102	0.367	85.62	94.23	62.90	96.71	60.68	80.03

**Table 3:** Comparison of computational cost.

Method	FLOPs (T)	#Params (M)	FPS
MLoRE [18]	1.453	509.58	7.61
MECO (ours)	1.034	416.14	11.20

**Table 4:** The effectiveness of the number of expert codebooks.

*N*	*k*	Abs Rel	mIoU (%)	FLOPs (T)	#Params (M)
5	3	0.110	79.57	1.034	400.35
10	6	0.108	79.60	1.034	408.25
15	9	0.102	80.03	1.034	416.14
20	12	0.099	79.68	1.034	422.46

**Table 5:** The effectiveness of the routing and codebook update method.

Routing	Codebook Update	Abs Rel	mIoU (%)
Soft	EMA	0.112	79.17
Top-*k*	EMA	0.103	79.15
Top-*k*	Dictionary loss	0.102	80.03

## Data Availability

The data presented in this study are available on request from the corresponding author.
